# Association Study between Coronary Artery Disease and *rs1333049* and *rs10757274* Polymorphisms at *9p21* Locus in South-West Iran 

**DOI:** 10.22074/cellj.2015.515

**Published:** 2015-04-08

**Authors:** Ali Mohammad Foroughmand, Emad Nikkhah, Hamid Galehdari, Mohammad Hossin Jadbabaee

**Affiliations:** 1 Department of Genetics, Shahid Chamran University, Ahvaz, Iran; 2 Department of Cardiology, Jondishapoor University of Medical Sciences, Ahvaz, Iran

**Keywords:** CAD, *9p21*, Polymorphism

## Abstract

**Objective:**

Coronary artery disease (CAD) is a multi-factorial and heterogenic disease with atherosclerosis plaques formation in internal wall of coronary artery. Plaque formation results to limitation of the blood reaching to myocardium leading to appearance of some problems, such as ischemia, sudden thrombosis veins and myocardial infarction (MI). Several environmental and genetic factors are involved in prevalence and incident of CAD as follows: hypertension, high low density lipoprotein-cholesterol (LDL-C), age, diabetes mellitus, family history of early-onset heart disease and smoking. According to genome wide association studies (GWAS), five polymorphisms in the *9p21* locus seem to be associated with the CAD. We aimed to evaluate the remarkable association of two polymorphisms at *9p21* locus, *rs1333049* and *rs10757274*, with CAD.

**Materials and Methods:**

This experimental study was conducted in Golestan, Aria Hospitals and Genetics Lab of Shahid Chamran University in the city of Ahvaz, Iran, in 2010- 2011. The collected blood samples belonging to 170 CAD patients (case group) and 100 healthy individuals (control group) were analyzed by tetra-primer amplification refractory mutation system (ARMS)-polymerase chain reaction (PCR) technique. The results were analyzed using software package used for statistical analysis (SPSS; SPSS Inc., USA) version 16. A value of p<0.05 and an odd ratio (OR) with 95% confidence intervals (CI) were considered significant.

**Results:**

The frequencies of CC, CG and GG genotypes for *rs1333049* polymorphism in patients were 18.2, 65.3 and 16.5%, while in controls, the related values were 25, 67 and 8%, respectively. GG genotypes of *rs1333049* polymorphism in CAD patients were more than control cases (OR: 0.354, 95%CI: 0.138-0.912, p=0.032). The frequencies of AA, AG and GG genotypes for *rs10757274* in CAD patients were 8.2, 58.3 and 33.5%, while in controls, the related values were 35, 63 and 2%, respectively. GG Genotype in *rs10757274* polymorphism in CAD patients was found more than control cases (OR: 0.014, 95% CI: 0.003 -0.065, p=0.0001).

**Conclusion:**

The *rs1333049* polymorphism at *9p21* locus shows a weak association with CAD, whereas *rs10757274* polymorphism reveals a significant association with CAD. These variants may help the identification of patients with increased risk for coronary artery disease.

## Introduction

Coronary artery disease (CAD) or coronary heart disease (CHD) is caused by atherosclerotic plaque formation in coronary arteries. The atherosclerotic plaque prevents blood from flowing through arteries. Approximately, 1.3 million Americans have shown CHD. The condition leading to heart attack and sudden death may be recognized in middle age or later with symptoms such as chest pain or angina. Progression of CAD in women before menopause is less than men of same age, whereas 10 years after menopause, the risk will be the same for both sexes. In addition, CAD is considered as a multi-factorial and heterogenic disease that are often associated with other heart diseases including high blood pressure (1,3). 

CAD progression often occurs during a ten-year period starting with lipid accumulation, scratching the lining, and finally, hardening of coronary arteries which leads to inflammation. By the blocking of 50 to 60% of coronary arteries, CAD symptoms can be appeared (3,4). 

The first description of the symptoms of CAD was introduced in 1768 (5). The factors involved in CAD disease are categorized into the following items: I. non-modifiable risk factors (age, gender and heredity), II. modifiable risk factors [hypertension, hyperlipidemia, smoking, diabetes (6,10), behavioral factors and left ventricular hypertrophy (LVH)] and III. protective factors ( high density lipoprotein-cholesterol (HDL-C), exercise and estrogen) (11,14). 

## Epidemiology of CAD and myocardial infarction (MI)

The cardiovascular diseases are the most common diseases worldwide. As in America, about 14 million adults show signs of CAD. One-third of the 1.5 million of the patients experiencing MI have died. The financial costs associated with CAD are approximately 50 to 100 billion dollars a year (15). As in Great Britain and the United States, CAD is the main cause of death in most countries. In addition, CAD mortality rate in Finland has showed that 500 of every 100,000 people with this disease lose their lives (16,17). 

## 9P21 locus

The genome-wide association studies (GWAS) have showed that *9p21* locus is associated with CAD (18). In addition, Wellcome Trust Case Control Consortium (WTCCC) and other studies conducted in German, Japan, Korea and Italy have considered *9p21* locus as a risk factor for CAD (18,22). 

Generally, at the *9p21* locus, several polymorphisms exist, but only five polymorphisms, *rs1333049*, *rs10757274, rs2383206, rs2383207* and rs10757278, have primary roles to predict CAD. 

The *rs1333049* have shown a good association with CAD because C allele of this single nucleotide polymorphism (SNP) was converted to G nucleotide that was first reported by Samani et al. (19) in the German population. 

Other polymorphisms of the *9p21* locus are *rs10757274* associated with CAD that was first reported by McPherson et al. (23) in the Canadian population. Then, many other studies have also confirmed the association of *rs10757274* SNP with CAD and showed A allele is changed to G allele (21,23,24). In this study, the association of two polymorphisms at *9p21* locus (*rs1333049*, *rs10757274*) with CAD was investigated in the population living in Southwest Iran. 

## Materials and Methods

In this experimental study, the controls and patients were chosen among individuals referred to the Angiography and the CT-Angiography Divisions of the Golestan and Aria Hospitals, Ahwaz, Iran, in 2010. This study was conducted on 170 CAD patients and 100 healthy controls. The study included patients who were 50 years or older and who were diagnosed with coronary artery stenosis (according to cardiology tests). Approximately, 5 ml of blood from both groups were collected in falcon tubes containing 0.5 ml of 0.5 ethylene diaminetetraacetic acid (EDTA) as anti blood clotting. This study was approved by the Ethical Committee of Ahvaz Jondishapoor University of Medical Sciences. A signed informed consent was obtained from all participants. 

## DNA extraction

DNA extraction was done using Diatom DNA prep 100 kit (Cinna gene Co., Iran). After that, DNA quality was tested by gel electrophoresis techniques. 

## Tetra-primer amplification refractory mutation system (ARMS)-polymerase chain reaction(PCR)

Tetra-primer ARMS-PCR is a rapid simple technique and has more advantages in comparison to other techniques such as restriction fragment length polymorphism (RFLP) (25). Tetra-primer ARMS-PCR was used to check polymorphism. In this technique, there are four primers of which two primers external and two primers are internal that are used instead of two primers in the conventional PCR. The optimal length of tetra ARMS primers is about 26-32 bases. Two round-trip primers are complementary to each other, while their concentration must be equal, about 50 pmol. We designed primers for both polymorphisms (*rs1333049* and *rs10757274*) using CLC Main Workbench (Qiagen, Denmark) (Table 1). 

## PCR thermal cyclers

In order to obtain the optimum temperature for PCR, a temperature gradient was carried out, and the PCR thermal cyclers were then performed as summarized in tables 2 and 3. 

## Observation of PCR products on agarose gel

PCR products of the *rs1333049* polymorphism (C/G) and of *rs10757274* polymorphism (A/G) were electrophoresed on 1.5 and 2% agarose gel, respectively, using 4 ml PCR products and 2 ml loading buffer for each sample. After electrophoresis, the gel was stained with ethidium bromide for 30 minutes and were observed by a ultra violet (UV) illuminator device (UV Tec CO., Russia). Moreover, according to the type of products observed on the gel, image polymorphisms genotype were determined. The products of each genotype are shown in tables 4 and 5. 

**Table 1:** Primers of tetra ARMS for 9p21 locus polymorphisms including rs1333049 and rs10757274


Type	Rs1333049 polymorphismSequence	CG%	Tm

**OF primer**	5´-CGAAGTAGAGCTGCAAAGATATTTGGAA-3´	39.3	62.2
**OR primer**	5´-GGGCTCATAATTGCTGAATAAAACAGAA-3´	35.7	60.7
**IF primer**	5´-CCTCATACTAACCATATGATCAACAGATG-3´	37.9	62.4
**IR primer**	5´-CTTACCTCTGCGAGTGGCTGCTTATG-3´	53.8	66.4
***rs10757274* polymorphism**			
**OF primer**	5´-TATGTAATGGCCTTCTTTGTCTCTTTTG-3´	35.7	60.7
**OR primer**	5´-AATGAATAAATGCTAACTTCTGCCTCAC -3´	35.7	60.7
**IF primer**	5´-GTGGGTCAAATCTAAGCTGAGTGTGGA -3´	48.1	64.9
**IR primer**	5´-ATCTATCTAGTGAATTTCAATTATGGCC -3´	32.1	59.2


ARMS; Amplification refractory mutation system, Tm; Melting temperature, OF; Outer forward, IF; Inner forward, OR: Outer reverse and IR; Inner reverse.

**Table 2:** The PCR thermal cyclers of the rs1333049 polymorphism


Stage	Number of repeat	Section	Temperature (˚C)	Time

**First**	1 cycle	First denaturation	95	5 minutes
		Secondary denaturation	95	45 seconds
**Second**	30 cycles	Annealing	62	90 seconds
		First extension	72	70 seconds
**Third**	1 cycle	Secondary extension	72	7 minutes


PCR; Polymerase chain reaction.

**Table 3:** The PCR thermal cyclers of the rs10757274


Stage	Number of repeat	Section	Temperature (˚C)	Time

**First**	1 cycle	First denaturation	95	5 minutes
		Secondary denaturation	95	45 seconds
**Second**	35 cycles	Annealing	52	90 seconds
		First extension	72	90 seconds
**Third**	1 cycle	Secondary extension	72	7 minutes


PCR; Polymerase chain reaction.

**Table 4:** Comparison of size and genotype of the rs1333049 polymorphism analyzed by gel electrophoresis


Productgenotype	422 bp(Product along with the external primers)	263 bp(C allele)	214 bp(G allele)

**CC**	+	+	-
**CG**	+	+	+
**GG**	+	-	+


PCR; Polymerase chain reaction.

**Table 5:** Comparison of size and genotypes of the rs10757274 polymorphism analyzed by gel electrophoresis


Product	412 bp	249 bp	218 bp
genotype	(Product along with the external primers)	(G allele)	(A allele)

**AA**	+	-	+
**AG**	+	+	+
**GG**	+	+	-


### Sequencing and analyzing of the samples

The obtained PCR products were compared in terms of length and categorized into several groups (Tables 4,5), while some samples were then prepared for sequencing. The results were analyzed using software package used for statistical analysis (SPSS, SPSS Inc., USA) version16. A value of p<0.05 and an odd ratio (OR) with 95% confidence intervals (CI) were considered significant. 

### Results

Average age values of patients and of healthy subjects are 58.66 ± 10.4 and 53.30 ± 7.2 years, respectively. The obtained results also showed that 89.4% of patients and 83% of healthy subjects were married. In addition, 35.9% of patients had family history of heart disease among whom 28.8% of their parents (patient group) had a consanguinity marriage. The ethnicity of participants was mainly Arabs living in city of Ahvaz, Khozestan Province, Southwestern Iran. 

Mean age of diabetic patients (28.2%) was 52.21 ± 4.3 years, while mean age of non-diabetics patients was 54.34 ± 5.4 years. The findings revealed that mean age of patient with high blood lipids (32.4%) is 52.94 ± 5.7 years, while none-high blood lipids patients show mean age of 53.91 ± 7.1. Mean age of patients with high blood pressure (44.7%) is 54.43 ± 4.9 years, while mean age of patients without high blood pressure is 52.06 ± 3.8. Mean age of smoker patients (24.7%) is 54.05 ± 4.1 years, while related value of non-smoker patients is 53.21 ± 5.5. 

## Tetra-primer ARMS-PCR products of rs1333049 and rs10757274 polymorphisms

**Fig.1:**
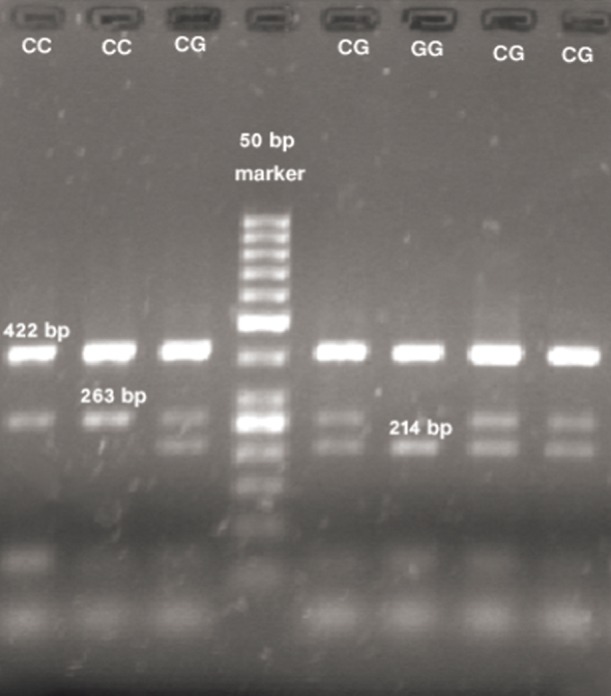
Electrophoresis results of PCR products of rs1333049 polymorphism. PCR; Polymerase chain reaction.

**Fig.2:**
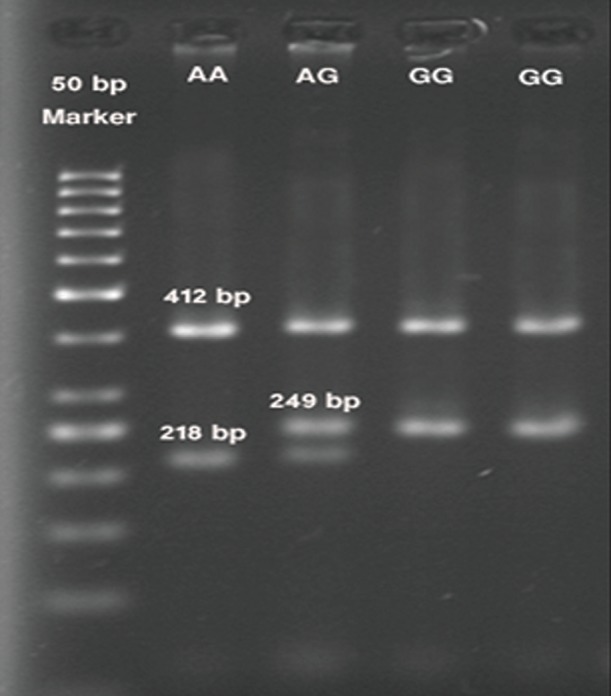
Electrophoresis results of PCR products of rs10757274 polymorphism. PCR; Polymerase chain reaction.

The genotype and size of bands on gel belonging to alleles of *rs1333049* and *rs10757274* polymorphisms were identified as shown in figures 1 and 2. The tables 4 and 5 also show the results of gel electrophoresis analysis. 

## Sequencing results

To confirm the tetra Arms PCR results, three samples of each polymorphism were sequenced. 

**Fig.3:**
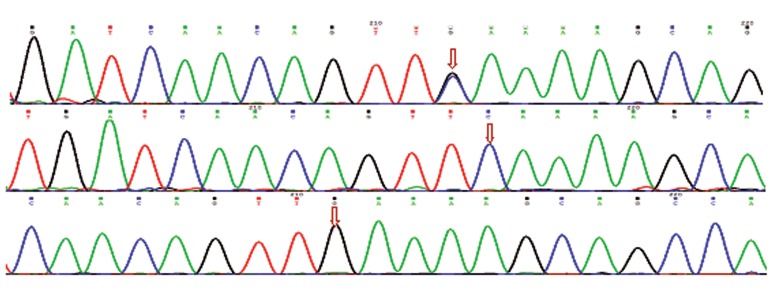
Sequencing analysis of rs1333049 polymorphism (arrows indicate CG, CC and GG genotypes, respectively).

**Fig.4:**
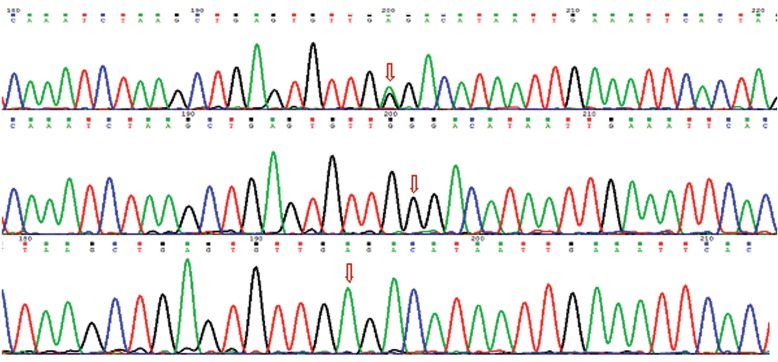
Sequencing analysis of rs10757274 polymorphism (arrows indicate AG, GG and AA genotypes, respectively).

The sequencing results confirmed tetra ARMS PCR results (Figs.3,4). 

## Statistical results of polymorphisms

After genotyping all samples of each polymorphism, the results were statistically analyzed (Tables 6,7). 

**Table 6:** Comparison of genotype frequencies of the rs1333049 polymorphism between two groups in order to verify its association with CAD


Genotype	Controls	Patients	OR	95% CI for OR		P value
				Low	High		

**CC**	25	31	0.806	-	-	-
**CG**	67	111	0.747	0.408	1.375	0.350
**GG**	8	28	0.354	0.138	0.912	0.032


CAD; Coronary artery disease, OR; Odd ratio and CI; Confidence intervals.

**Table 7:** Comparison of genotype frequencies of the rs10757274 polymorphism in order to verify its association with CAD


Genotype	Controls	Patients	OR	95% CI for OR		P value
				Low	High	

**AA**	35	14	2.500	-	-	-
**AG**	63	99	0.255	0.127	0.510	0.0001
**GG**	2	57	0.014	0.003	0.065	0.0001


CAD; Coronary artery disease, OR; Odd ratio and CI; Confidence intervals.

## The overall result

The *rs1333049* polymorphism showed a poor association (X2=4.343, df=1; p value=0.032), while the *rs10757274* polymorphism showed a strong association with CAD (X2=53.476, df =1; p value=0.0001).

## Discussion

Comparison of the obtained results between other studies and the present study is given in table 8 (18-21, 23, 24, 26).

As our results indicate, presence of a group of risk factors, such as family history of heart disease, consanguineous marriage, age, diabetes mellitus, smoking, hypertension and high blood lipid, increases incidence of CAD.

The average age of onset of CAD in patients with family history of heart diseases (51.54 ± 3.6 years) was lower in comparison with patients without family history (55.48 ± 4.2 years). It was also suggested that the family history of heart diseases may predispose individual to CAD. As supposed, the average age of onset of CAD in patients whose parents had consanguinity marriage was lower by more than 2 years in comparison with patients whose parents were not relative. Our results also show diabetes, high triglycerides and low density lipoprotein cholesterol (LDL-C) increase the risk factors of CAD. High blood pressure and smoking are the other risk factors, whereas this study did not confirm the effects of these two factors on CAD.

The present study showed a weak association between *rs1333049* polymorphism and CAD (p=0.032) as reported by Shen et al. (21), while a good association was observed between CAD and *rs1333049* in Arab patients (p=0.023), which is consistent with the studies by Samani et al. (19), Hinohara et al. (18) and Schunkert et al. (20). The difference in our results may be due to racial or life styles differences of Arab ethnic group.

**Table 8:** Comparison of the other studies results with the present study


Polymorphism	Source	P value	Country

**Rs1333049**	Samani et al. (19)	0.0001	Germany
	Hinohara et al. (18)	0.00027	Japan and South Korea
	Schunkert et al. (20)	0.0079	Germany and UK
	Shen et al. (21)	No association	America
	Present study	0.032	Iran
**Rs10757274**	McPhersson et al. (23)	<0.025	Canada
	Shen et al. (21)	0.037	Italy
	Talmud et al. (24)	0.01	UK
	Dehghan et al. (26)	No association	Netherlands
	Present study	0.0001	Iran


## Conclusion

The present study showed a significant association between *rs10757274* polymorphism and CAD (p=0.0001). This polymorphism may be a marker for the diagnosis of CAD, but more research is needed to reveal existence of involved genes in CAD prevalence.
